# Division of the Mucous Membrane to Cure Fissures of the Rectum

**Published:** 1855-01

**Authors:** 


Division of Mucous Membrane to cure Fissures of the Rectum.—Mr. Quain, in his recent work on Diseases of the Rectum, states that while performing Boyer's operation, viz: division of the sphincter ani for fissure of the rectum, his patient moved so that the bistoury only divided the skin and mucous membrane. In watching the effects of this simple operation, he found the success was complete and the patient cured. In claiming this agreeable modification in the treatment of fissures of the rectum, he subsequently found he had been anticipated in it by Sir Benj. Brodie.—*Nashville Journal.*

